# Royal Medical and Chirurgical Society

**Published:** 1898-10-29

**Authors:** 


### ROYAL MEDICAL AND CHIRURGICAL SOCIETY.

From an epidemiological point of view nothing could be more important than the paper read on Tuesday evening last before the Royal Medical and Chirurgical Society by Dr. Lambert Lack, on the subject of Fibrinous Rhinitis and its Relation to Diphtheria. We need not here stay to describe the symptoms of the disease which may shortly de defined as a subacute or chronic affection of the nose, characterised by the formation of a fibrinous, membranous. or shreddy exudation on the nasal mucous membrane, It is necessary, however, to insist upon the comparative triviality of the general symptoms of the malady when contrasted with what occurs when true diphtheria attacks the nose, for it is the general experience that nasal diphtheria is the most fatal of all the varieties of the disease. Moreover, while in the cases which survive the latter, paralytic affections are common and severe, none were observed in the cases of membranous rhinitis which were the subjects of this paper, although they were regularly sought for during two to three months. We have then, two diseases, which so far as their clinical symptoms are concerned, are absolutely distinct from one another. Bacteriologically, however, the difference between these diseases is not so obvious, for on examination it was found that in every case an abundant growth of the typical Klebs-Loeffler bacillus was obtained. In 15 cases the bacillus was found in apparently pure culture, while in 18, other organisms were found along with it. Moreover, these bacilli were shown by experiments upon animals to be in a state of considerable virulence. Again, the toxins produced by the organisms taken from cases of membranous rhinitis were found to be neutralised by the anti-toxin of diphtheria, so that, taking it altogether, it is clear not only that the bacilli are identical, but that the mildness of the affection under consideration does not depend upon any lessened virulence or toxin-producing power of the organisms found in it. As control experiments, Dr. Lack then proceeded to investigate the condition, in regard to diphtheria bacilli, of other throats and other noses, with the result of showing that the Klebs-Loeffler bacillus is found commonly in the normal throats of healthy people who have not been exposed to diphtheritic infection, although not quite so commonly as in those who have been exposed to it; that the bacillus is found just as frequently in cases with normal throats as in cases with the non-diphtheritic angina; that the bacillus commonly occurs in the healthy nose, and is found in about 25 per cent. of all cases with any form of nasal discharge. Added to all this is a point which must be insisted on, namely, that, although the specific bacillus of diphtheria was found in every case of membranous rhinitis, in not a single instance of the latter disease was it possible to show that cases of unmistakable diphtheria had occurred in connexion with it. What, then, about the bacteriological diagnosis of diphtheria, the institution of public laboratories, and all the rest of it?

